# “This is where it all started” – the pivotal role of PLCζ within the sophisticated process of mammalian reproduction: a systemic review

**DOI:** 10.1186/s12610-017-0054-y

**Published:** 2017-05-21

**Authors:** Itai Gat, Raoul Orvieto

**Affiliations:** 1 IVF Unit, Department of Obstetrics and Gynecology, Sheba Medical Center, Tel Hashomer, Israel; 2 Sackler Faculty of Medicine, Tel Aviv University, Tel Aviv, Israel; 3 Pinchas Borenstein Talpiot Medical Leadership Program, Sheba Medical Center, Tel Hashomer, Israel; 4 The Tarnesby-Tarnowski Chair for Family Planning and Fertility Regulation, Sackler Faculty of Medicine, Tel Aviv, Israel

**Keywords:** Gametogenesis, Oocyte activation, Calcium oscillations, Embryogenesis, PLCζ, IVF, ICSI, Male infertility, Gamétogenèse, Activation ovocytaire, Oscillations calciques, Embryogenèse, Phospholipase C zeta (PLCζ), FIV, ICSI, Infertilité masculine

## Abstract

Mammalian reproduction is one of the most complex and fascinating biological phenomenon, which aims to transfer maternal and paternal genetic material to the next generation. At the end of oogenesis and spermatogenesis, both haploid gametes contain a single set of chromosomes ready to form the zygote, the first cell of the newly developing individual. The mature oocyte and spermatozoa remain in a quiescent state, during which the oocyte is characterized by nuclear and cytoplasmic arrest, while the spermatozoa necessitates further maturation within the epididymis and female reproductive track prior to egg fertilization. Either in vivo or in vitro, the sperm initiates a series of irreversible biochemical and physiological modifications in the oocyte. The earliest detected signal after fertilization is cytosolic Ca^2+^ oscillations, a prerequisite step for embryo development. These oscillations trigger the release of the oocyte from the second meiosis arrest towards embryogenesis, also known as “oocyte activation”. Phospholipase C zeta (PLCζ) is a unique sperm-soluble protein responsible for triggering the InsP_3_/Ca^2+^ pathway within the oocyte, leading to Ca^2+^ oscillations and consequently to embryo development. The specific structure of PLCζ (compared to other PLCs) enables its specialized activity via the preserved X and Y catalytic domains, as well as distinct features such as rapid onset, high sensitivity to Ca^2+^ and cession of oscillations upon zygote formation. The emerging discoveries of PLCζ have stimulated studies focusing on the possible clinical applications of this protein in male infertility evaluation and management during IVF/ICSI. Fertilization failure is attributed to lack of oocyte second meiosis resumption, suggesting that ICSI failure may be related to impaired PLCζ activity. Microinjection of recombinant human PLCζ to human oocytes after ICSI fertilization failure may trigger Ca^2+^ oscillations and achieve successful fertilization, offering new hope for couples traditionally referred to sperm donation. However, more studies are still required prior to the routine implementation of this approach in the clinic. Directions for future studies are discussed.

## Résumé

La reproduction des mammifères est l’un des phénomènes biologiques les plus complexes et fascinant; son objectif est de transférer le matériel génétique maternel et paternel à la génération suivante. A la fin de l’ovogenèse et de la spermatogenèse, les deux gamètes haploïdes contiennent un ensemble unique de chromosomes prêts à former un zygote, la première cellule du nouvel individu en développement. L’ovocyte mature et les spermatozoïdes restent dans un état quiescent au cours duquel l’ovocyte est caractérisé par un arrêt cytoplasmique et nucléaire, alors que les spermatozoïdes ont besoin d’une maturation ultérieure dans l’épididyme et le tractus génital femelle avant de pouvoir féconder l’ovocyte. Que ce soit in vivo ou in vitro, le spermatozoïde amorce une série de modifications biochimiques et physiologiques dans l’ovocyte.

Le premier signal détecté après la fécondation est constitué des oscillations Ca2+ cytosoliques, une condition préalable au développement embryonnaire. Ces oscillations libèrent l’ovocyte de son arrêt en seconde méiose vers l’embryogenèse, phénomène connu sous le nom ‘d’activation de l’ovocyte’. L’isoforme phospholipase C zeta (PLCζ) est la seule protéine soluble du spermatozoïde capable d’activer dans l’ovocyte la voie de signalisation inositol triphosphate/Ca2+ qui mène aux oscillations Ca2+ et par conséquent au développement de l’embryon.

Par rapport aux autres PLC, la structure spécifique de PLCζ lui confère une activité spécialisée via les domaines catalytiques préservés de X et Y, ainsi que des caractéristiques propres tels un déclenchement rapide, une grande sensibilité au Ca2+ et un arrêt des oscillations à la formation du zygote. Les découvertes récentes de PLCζ ont induit des études centrées sur les possibles applications cliniques de cette protéine dans l’évaluation et la prise en charge de l’infertilité masculine lors de FIV/ICSI. L’échec de fécondation est attribué à l’absence de reprise de la seconde méiose ovocytaire, suggérant la possibilité que l’échec de l’ICSI puisse être lié à une activité défectueuse de la PLCζ dans le spermatozoïde. La micro injection de PLCζ humaine recombinante dans des ovocytes humains après échec de fécondation en ICSI pourrait déclencher des oscillations Ca2+ et permettre une fécondation réussie, offrant de nouveaux espoirs aux couples traditionnellement orientés vers le don de spermatozoïdes. Il est toutefois nécessaire de disposer d’un plus grand nombre d’études avant toute mise en œuvre de cette approche en clinique. Des orientations pour de futures études sont discutées.

## Backgroud

Mammalian reproduction is one of the most complex and fascinating biological phenomenon, aimed at transfering maternal and paternal genetic material to the next generation. Our understanding regarding the molecular and cellular phases of fertilization is rapidly progressing in the milieu of assisted reproductive technology (ART) [1]. Within this highly sophisticated process, starting from gamete development and ending with newborn delivery, specific attention is placed on the process of fertilization. Factors involved in the primary interaction between sperm and egg have been the focus of numerous studies over the past decades. This review focuses on the sperm protein phospholipase C zeta (PLCζ), a unique and key protein which regulates the initiation of the sperm-egg interaction, within the general perspective of mammalian reproduction. The emerging understanding of the crucial role of PLCζ in oocyte activation is primarily rooted in basic science research and bear substantial potential for possible future clinical applications. The aim of this review was to provide an overview of the current knowledge regarding the structure and function of PLCζ within the context of the sophisticated process of mammalian reproduction and to suggest future research approaches, mainly focusing on its clinical applicability. The systematic literature search queried PubMed for the keywords “PLCζ, “PLC zeta” and “oocyte activation”.

## Gametogenesis

Gametogenesis, i.e., the development and formation of mature oocyte and spermatozoa through meiosis, is a fundamental and unique process occurring within the ovaries and testicles, respectively [2]. Oogenesis and spermatogenesis share similar features, accompanied by multiple basic differences. In humans, embryonic primordial germ cells (PGC) derived from the genital ridge, serve as the source for germ cell formation, which then differentiate into oogonium or alternatively to spermatogonial stem cell (SSC), depending on the presence of the SRY gene [3]. The mature spermatozoa complete meiosis within the testicular seminiferous tubules, while the egg completes the first meiosis and starts the second meiosis just prior to ovulation. Second meiosis is arrested in metaphase and only resumes in case of fertilization [2]. At the end of oogenesis and spermatogenesis, the mature oocyte and spermatozoa remain in a quiescent state: the oocyte is characterized by nuclear (mainly chromosomal segregation) and cytoplasmic (RNA, protein and mitochondrial accumulation) block [2], and testicular haploid spermatozoa necessitate further maturation within the epididymis and female reproductive track prior to egg fertilization [4, 5].

## In vivo and in vitro fertilization

In vivo human fertilization involves various paracrine, ion-regulated and other modes of egg-sperm cross-talk. Specific molecules secreted by the oocyte and cumulus cells mediate sperm hyperactivation, chemotaxis and acrosome reaction [6–8]. In vitro fertilization (IVF) is an ART aimed to achieve pregnancy among infertile couples after failure to achieve in vivo fertilization and implantation. The lack of a natural female reproductive environment during IVF and intracytoplasmic sperm injection (ICSI), requires artificial sperm preparation to induce sperm maturation prior to fertilization [9].

Male factor is a major culprit of infertility, accounting for up to 50% of infertile couples [10]. While some couples with male infertility may conceive following intrauterine insemination (IUI), those with severely impaired semen analysis and those with repeated IUI failures are commonly referred to IVF accompanied by ICSI. The introduction of ICSI [11] was a milestone and presented a revolution in the management of male infertility, since it enables biological offspring in cases of severe oligospermia and azoospermia [12, 13], which previously required a couple to pursue sperm donation. While conventional IVF includes incubation of sperm with the retrieved oocyte surrounded by cumulus cells, and requires intact acrosome reaction during fertilization, cumulus cells are detached from the egg prior to ICSI, enabling direct injection of a single sperm into the MII oocyte [11].

## Physiological mechanisms involved in egg activation

Immediately following fertilization, either in vivo or in vitro, the sperm initiates a series of irreversible biochemical and physiological oocyte modifications, which rescue it from predestined apoptosis [14]. The earliest detected fertilization signal is a distinctive series of cytosolic Ca^2+^ oscillations, a prerequisite step for embryo development [15]. These oscillations release the oocyte from the MII phase arrest and initiate embryogenesis via meiotic resumption, cortical granule exocytosis, sperm nucleus decondensation, recruitment of maternal mRNA and pronuclear development – collectively known as “oocyte activation” [16, 17]. These processes involve multiple protein kinases, which are responsible for conveying Ca^2+^ oscillation cues to achieve essential activation events, such as cytoskeletal reorganization and formation/extrusion of the second polar body, collectively leading to embryo development [18].

The role of intracellular Ca^2+^ and inositol 1,4,5-triphosphate (InsP_3_) within the fertilized eggs has been at the forefront of recent research [19]. That signal transduction pathway involved in many biological processes, is first initiated by phospholipase C (PLC) isoforms, which hydrolyze the precursor phosphatidylinositol 4,5-biphosphate (PIP_2_), resulting in the formation of InsP_3_ and diacylglycerol (DAG). Consequently, InsP_3_ binds the InsP_3_ receptor in the endoplasmic reticulum (ER), which stimulates the release of Ca^2+^ from intracellular storage [20]. However, the specific link between sperm-egg interaction and increased InsP_3_ production has been debated. Initial studies relied on the predominant hypothesis that the sperm activates the egg via binding to the egg membrane. Only later, researchers demonstrated that InsP_3_ production and subsequent Ca^2+^ oscillations are triggered by a sperm-derived soluble protein which is released to the oocyte cytoplasm immediately after sperm-egg fusion [19, 21].

## Sperm-derived protein PLCζ

Evidences of InsP_3_/Ca^2+^ pathway involvement, alongside the high level of PLC activity measured in sperm extracts, have suggested the participation of PLC isoform in oocyte activation [22]. Saunders et al. [23] reported for the first time that PLC﻿zeta (ζ) isoform in mouse, is an essential soluble protein responsible for triggering InsP_3_/Ca^2+^ pathway, followed by Ca^2+^ oscillations and eventually oocyte activation and embryo development. PLCζ cloning demonstrated a relatively small 74-kDa structure compared to other PLC isoforms. Their most fascinating finding was Ca^2+^ oscillations triggered by microinjection of PLCζ complementary RNA (cRNA) into MII mouse oocytes as shown in sperm fertilization, resulting in embryo development up to blastocyst stage. Interestingly, injection of mRNA of PLCδ1, the PLC isoform most similar to PLCζ, failed to induce Ca^2+^ oscillations, confirming the unique structure and function of PLCζ, [23]. Later on, Cox et al. isolated and characterized the 70-kDa human PLCζ (hPLCζ), and reported that the PLCζ gene is located on chromosome 12, composed of 15 exons and is solely expressed within the testicular tissue. Microinjection of cRNA of the hPLCζ to mouse oocytes resulted in Ca^2+^ oscillations and blastocyst formation. The authors noted high structural homogeneity (>80%) and identical function between human, simian and mouse PLCζ and its identical function, emphasizing the preserved PLCζ expression throughout mammalian reproduction [24]. Further studies have shown PLCζ structure specificity in different mammalian species [25].

## PLCζ mechanism of action

**Fig. 1:**
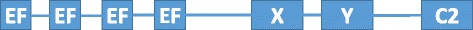
Schematic diagram of PLCζ domains. The most conserved region among PLCs is the XY linker region, which hydrolyses PIP_2_, which triggers the InsP_3_/Ca^2+^ pathway [26]. The EF regions, which enhance PLCζ stability and Ca^2+^ binding, significantly contribute to the high PLCζ sensitivity to Ca^2+^ [16]. Moreover, EF domains binding to the C2 region, bend the PLCζ to a specific configuration, which exposes the catalytic X and Y domains and enables efficient PIP_2_ hydrolysis [27]. The XY-linker domain is another key regulator of the catalytic domains

Several studies reported a correlation between specific PLCζ structural characteristics and its unique ability to provoke Ca^2+^ oscillations within the eggs even at very low concentrations [26]. At the same time, it shares numerous domains with other PLC isoforms such as four EF hand regions, X and Y catalytic domains (separated by the XY-linker domain) and a C2 domain [24] (Fig. 1). The most conserved region among PLCs is the X-Y catalytic region, which hydrolyses PIP_2_, which then triggers the InsP_3_/Ca^2+^ pathway [26]. The EF regions, which enhance PLCζ stability and bind Ca^2+^, contribute significantly to the high sensitivity of PLCζ to Ca^2+^ [16]. Moreover, EF domain binding to the C2 region is responsible for the protein’s specific configuration, which exposes the catalytic X and Y domains and permits efficient PIP_2_ hydrolysis [27]. The XY-linker domain is another key regulator of the catalytic domains by two suggested mechanisms. First, nuclear localization signals within this region drive PLCζ translocation upon zygote interphase, thereby modulating its activity and preventing excessive PIP_2_ hydrolysis [28]. Second, its positive charge, opposed to the negative charge in other PLC isoforms, enable efficient anchoring of PLCζ to the negatively charged PIP_2_ [29]. On the other hand, PLCζ selectively lacks the plextrin-homology (PH) domain at the N terminal which is consistently expressed in other PLC isoforms [24]. The PH domain is a key factor in PLC binding to the plasma membrane such as PIP_2_ and G proteins [30]. Consequently, PLCζ activity is characterized by cytosolic activity. Shortly after sperm-egg fusion, PLCζ diffuses through the egg cytoplasm and binds to intracellular vesicles containing PIP_2_, possibly via an egg-specific protein, resulting in massive PIP_2_ release and InsP_3_ production [21]. In conclusion, the distinctive structure of PLCζ enables activation of the InsP_3_/Ca^2+^ pathway by the preserved X and Y catalytic domains, with specific features such as rapid onset, high sensitivity to Ca^2+^ and cession of oscillations upon zygote formation.

Aside from its structure, PLCζ localization within the spermatozoa may have an impact on its function. However, its localization within sperm is not uniform throughout species [31]. Among human samples, distinct populations of PLCζ have been identified in the acrosomal, equatorial and post-acrosomal regions of the sperm head, with additional potential tail localization [32, 33], as demonstrated in other species [34–36]. Furthermore, variable PLCζ localizations before versus after capacitation have been reported in human samples [37], mouse and hamsters [38]. The underlying reasons for this variability are yet to be determined. It is reasonable to assume that specific localization impacts PLC solubility [31] and function, which may challenges the implementation of PLCζ-focused treatments in clinical settings.

## Alternative sperm factor post-acrosomal WW-domain binding protein and assisted oocyte activation techniques

Post-acrosomal WW-domain binding protein (PAWP), located in the post-acrosomal sheath region of the perinuclear theca, is another sperm protein suggested to have essential role in oocyte activation. Similar to PLCζ studies, RNA or recombinant protein injections resulted in oocyte activation in multiple species including human [39, 40]. Consequently, PAWP has been suggested as an alternative sperm factor inducing embryogenesis independent of or in combination with PLCζ. However, the mechanism of action has not been established [41]. Furthermore, doubts have been raised regarding the capacity of PAWP to hydrolyze PIP_2_ and to initiate oocyte activation in vitro [42], as well its solubility due to its specific localization in the sperm head [31]. Therefore, Anifandis et al have stated that PLCζ has more grounds compared to PAWP due to its wider scientific support [41].

Since fertilization failure in ART is mostly attributed to absence of oocyte activation, several methods have been proposed to activate the eggs. Borges et al. reported the use of a calcium ionophore to induce single Ca^2+^ peak by massive cations influx [43]. Others have proposed electrical current-induced [44–46] or specific mechanical manipulation approaches [47]. A recent meta-analysis of randomized controlled trials concluded that currently there is not sufficient data to support either methodology in cases of fertilization failure or “rescue ICSI” [48]. Obviously, none of these techniques present the physiological Ca^2+^ oscillations. Therefore, RNA- or rhPLCζ-based methodologies, which have been demonstrated to trigger Ca^2+^ oscillations, seem promising modalities for improving fertilization rates after ICSI, especially in cases of fertilization failure.

## Clinical applications of PLCζ

The emerging discoveries regarding PLCζ have sparked studies focused on the possible clinical applications of this protein in male infertility evaluation and management. As described above, ICSI is a well-established treatment for male infertility which triggers Ca^2+^ oscillations and results with fertilization rate of approximately 70% [49, 50]. Although the prevalence of fertilization failure following ICSI is low, if encountered, the ICSI cycle is cancelled, incurring serious economic and psychological burdens [51, 52]. Importantly, most non-fertilized eggs never resume the second meiosis [53], suggesting egg activation failure as a possible culprit. Moreover, several sperm defects have been reported to be associated with ICSI failure [54]. All these findings suggest the possibility that ICSI failure may be sperm-related, possibly due to impaired PLCζ activity.

The crucial importance of PLCζ has led to numerous assessments of its applicability as a **diagnostic** tool for male infertility. Patients’ populations varied including those with globozoospermia (morphological sperm defect characterized by round sperm heads) or with low/failed fertilization [55]. Moreover, various methodological approaches have been used such as quantitative PLCζ measurements, morphological evaluations, analysis of localization within the spermatozoa and genetic assessments [32, 55–58]. More physiological approaches included microinjection of human sperm into mouse oocytes to evaluate sperm activation capacity [59]. Collectively, these reports raised various results regarding the diagnostic value of PLCζ in male infertility cases (Table 1). Interestingly, Kashir et al. recently presented an immunocytological analysis of sperm PLCζ, using a specific and innovative polyclonal antibody [60]. Their reported methodology may become a reliable evaluation for PLCζ visualization over time.

**Table 1:** Investigations focused on the diagnostic value of PLCζ in human sperm

Reference	Population	PLCζ related Findings
Yoon et al., 2008 [58]	Recurrent failed ICSI	Absent
Grasa et al., 2008 [37]	Fertile participants	Variable localization in uncapacitated and capacitated sperm
Heytens et al., 2009 [32]	Infertile patients	Low expression or abnormal forms
Yelumalai et al., 2015 [57]	Infertile patients	Low expression and specific localization patterns
Escoffier et al., 2015 [56]	Globozoospermia	Absent or extremely reduced expression

In daily clinical practice, ICSI is often the last treatment option in cases of male infertility, when aiming to achieve fertilization and biological parenthood. Following ICSI fertilization failure, couples are commonly referred for sperm donation. The aforementioned observations have led Yoon et al. to perform ICSI in mouse MII oocytes with human sperm in cases of ICSI fertilization failure, resulting with sperm inability to initiate Ca^2+^ oscillations. Furthermore, sperm samples of individuals with ICSI failure had presented no sperm PLCζ, as assessed by both immunofluorescence and Western blotting [58]. While Yoon et al. did not isolate any specific gene mutation, later reports hypothesized that a specific mutations, possibly of maternal origin, in the PLCζ gene may result in male infertility: PLCζ^H233L^ and PLCζ^H398P^ describe specific substitutions of leucine and proline amino acids, respectively, with histidine within the X and Y catalytic domains, respectively. Both mutations resulted in an abnormal PLCζ 3D structure, leading to impaired oocyte activation and infertility [29, 33, 61]. Escoffier et al. recently identified a missense mutation on PLC*ζ,* c.1465A > T, located in exon 13, changing an Ile at position 489 into a Phe (Ile489Phe) among two brothers and their respective wives, who experienced oocyte activation failure in the presence of normal semen analysis [62]. Furthermore, Ferrer-Vaquer et al. reported another heterozygosis mutation affecting the X catalytic domain and also emphasized that polymorphism within the PLCζ may play a role in its activation capability, even in the presence of normal semen analysis and among sperm donors [63]. These discoveries have further expanded our understanding of PLCζ-targeted clinical evaluations.

The next obvious step after PLCζ-focused investigations is to seek for innovative PLCζ–based **treatments** to couples with fertilization failure. Most PLCζ functions in mammalian species, including human, have been investigated by microinjection of mRNA or recombinant protein into mouse MII oocytes [23, 24, 64]. Importantly, Rogers et al. injected various concentrations of hPLCζ mRNA to human oocytes after fertilization failure following IVF/ICSI, and demonstrated successful triggering of Ca^2+^ oscillations, comparable to the pattern shown following successful IVF or ICSI [64, 65]. Yet, in the clinical setting, injected mRNA may be converted to cDNA by reverse transcriptase and then incorporated into the embryo genome [66], therefore recombinant protein should be preferred. A pioneering study introduced non-purified recombinant wild-type hPLCζ produced by transformed human embryonic kidney cells, which induced mouse oocyte activation upon injection [67]. One year later, Yoon et al. reported the injection of recombinant hPLCζ (rhPLCζ) into vitro matured human MII oocytes (without sperm injection), which resulted with the formation of a single PN the next day and two cell embryo within 48 h. Embryo haploidy was confirmed by FISH. The group also injected rhPLCζ into MII oocytes which failed to produce 2PN after ICSI, and achieved 2PN in 5/8 (62.5%) oocytes [68]. Their report verified, for the first time, the concept of “rescue PLCζ”, which encompasses analog perception as “rescue ICSI” after fertilization failure by conventional IVF. Importantly, the oocyte response to rhPLCζ, as measured by Ca^2+^ oscillations, varied significantly between patients, emphasizing the important role of oocyte quality in addition to male-related PLCζ activity [68]. Similarly, Nomikos et al. demonstrated human oocyte activation by Ca^2+^ oscillations, after injection of purified rhPLC; however, further embryo development was not reported [69]. In conclusion, microinjection of purified hPLCζ may provide hope for several patient populations. This innovative “rescue PLCζ” approach may be used immediately after ICSI fertilization failure after exclusion of 2PN appearance. RhPLCζ can also be microinjected with spermatozoa after history of ICSI fertilization failure, especially in couples with repetitive failures who were traditionally referred to sperm donation. However, more studies are needed prior to the routine clinical implementation of these approaches.

## Future directions

The presented studies supply comprehensive data regarding factors driving fertilization and provide ground for further related basic and clinical research. Firstly, novel gene mutations should be investigated, especially in cases of unexplained infertility or repetitive low fertilization rates when applying IVF/ICSI. It is reasonable to hypothesize that mutations within the non-catalytic domains (such as the XY linker and EF domains) may not necessarily lead to complete fertilization failure, but, rather to impaired PLCζ function resulting in decreased fertilization rates. Second, since PLCζ is specifically expressed in testicular tissue, further characterization during male germ cell differentiation may shed new light on this sophisticated process. It is reasonable to assume that PLCζ is expressed during the final differentiation stages, similar to acrosome and protamine, which are crucial for physiological spermatozoa function as well [70]. That aspect may be even more interesting in cases of round spermatid injection (ROSI) to oocytes during ART, which has been reported after failure to find mature spermatozoa in testicular biopsy [71]. Although ROSI may theoretically be suggested in cases of failure to extract spermatozoa, their fertilization potential is considered to be low due to lack of oocyte activation. Therefore ROSI should be performed with assisted oocyte activation (AOA), such as calcium ionophores [71].

The data which has accumulated over the last 15 years of infertility research, support future clinical directions for both the evaluation and treatment of male infertility, particularly with regard to application of rhPLCζ. Studies focusing on the physiological role of PLCζ, such as its quantitative expression in single spermatozoa [41] as well as the possible association between increased PLCζ and its colocalization with other proteins related to acrosome reaction and capacitation [1], may expand our understanding of its diagnostic potential. Furthermore, evaluation of PLCζ RNA and protein expressions among couples with unexplained infertility and/or ICSI failure may provide additional insights into its function and therapeutic potential. Newly discovered mutations may be of both diagnostic and prognostic significance. “Rescue PLCζ” after failed ICSI warrants further investigation in terms of safety and its potential impact on embryo development. This approach seems promising, particularly following the recent report of Sanusi et al., who reached satisfactory embryo development up to blastocyst stage [72]. In parallel, the impact of co-microinjection of sperm with rhPLCζ should be compared to conventional ICSI in couples with history of failed ICSI failure, with hopes to improve fertilization rates.

Additional investigations should focus on potential female factors, since successful fertilization is not only based on adequate spermatozoa but also on oocyte quality [51]. Oocyte maturation involves both nuclear (mainly chromosome-related) and cytoplasmic events, the latter involving multiple and parallel processes, such as increased Ca^2+^ within the ER and accumulation of mRNA and proteins required for early embryo development [2]. Therefore, the impact of rhPLCζ injection on various female factor-related infertility (age, PCOS, endometriosis etc.) should also be evaluated.

## Conclusions

In conclusion, successful gametogenesis and germ cell maturation are prerequisite for mammalian fertilization. PLCζ introduction into the egg cytoplasm leads to intense signal transduction events and early embryo development. The emerging data regarding PLCζ function has been primarily obtained in the mouse model and initial results regarding the impact of rhPLCζ injections into human oocytes are promising. RhPLCζ injection may provide an innovative solution for multiple patient populations, such as cases of ICSI fertilization failure within the same cycle after exclusion of 2PN appearance, or adjunct to ICSI after ICSI fertilization failure in previous ART cycles. HPLCζ may also prove effective in improving the currently low fertilization rate of ROSI. However, this technology is still far from routine usage, and will require performance of critical clinical studies.

## Abbreviations

ART: Assisted reproductive technology
DAG: Diacylglycerol
ER: Endoplasmic reticulum
ICSI: Intracytoplasmic sperm injection
InsP_3_: Inositol 1,4,5-triphosphate
IUI: Intrauterine insemination
IVF: In vitro fertilization
PGC: Primordial germ cells
PIP_2_: Phosphatidylinositol 4,5-biphosphate
PLC: Phospholipase C
rhPLCζ: Recombinant human PLCζ
ROSI: Round spermatid injection
SSC: Spermatogonial stem cell
